# The imperative for health promotion in universal health coverage

**DOI:** 10.9745/GHSP-D-13-00164

**Published:** 2014-02-11

**Authors:** Gloria Coe, Joy de Beyer

**Affiliations:** a U.S. Agency for International Development, Washington, DC, USA; b World Bank Institute, Washington, DC, USA

## Abstract

Health promotion and disease prevention have huge impact on health, yet given low priority, risk being overlooked in universal health coverage efforts. To effectively prioritize promotion and prevention, strong cadres of personnel are needed with expertise in legislation and health policy, social and behavior change communication, prevention and community health, health journalism, environmental health, and multisectoral health promotion.

Universal health coverage (UHC) centers on delivering effective, affordable health care, and the policy focus is often heavily on curative care. Health promotion, on the other hand, centers on keeping people well, largely through promoting healthy behavior and environments. Implementing robust, effective evidence-based health promotion programs would improve people's health profoundly and also help ensure the financial viability of UHC. The emerging importance of noncommunicable diseases and injuries (NCDIs) in the developing world increases the imperative for prevention and health promotion. Fortunately, vibrant examples with strong impact are emerging both in the West and in developing countries.

Health promotion programs implement a broad array of interventions to ensure optimal health and to prevent illness across the life span, at national, provincial, and community levels, involving multiple sectors of Government, notably Ministries of Education, Finance, Transportation, and Communication. Successful health promotion programs rely on qualified professionals specialized in areas as diverse as policy analysis, legislation, social psychology, social and behavior change communication, economics, sociology, and health journalism.

This article advocates that national policy and decision-makers should rebalance efforts in the health field to do far more to promote health and prevent disease. This will require: raising competencies, profiles, and incentives for health promotion and disease prevention professionals; a stronger health promotion curriculum in schools of public health and far more attention to health promotion and disease prevention in the training of doctors and other health care providers; and an improved legal, operational, and management framework of health promotion units in health ministries at national and provincial levels, with clearer roles and responsibilities and adequate budgets.

## THE DILEMMA OF UNIVERSAL HEALTH COVERAGE — HEALTH VS. HEALTH CARE

Global health faces a major dilemma. On the one hand, the World Health Organization (WHO) defines health as a “complete state of physical, mental and social well-being.”^1^ On the other hand, universal health coverage tends to focus on health “care” and health “services,” often in the context of health “insurance.”^2^ That leads to an emphasis on curative care. Ironically, health promotion generally takes a back seat, despite its enormous importance for well-being. We assert health promotion should be front and center. And the surging rates of NCDIs in developing countries only strengthen the need for health promotion.

Universal health coverage tends to focus on curative care without enough focus on health promotion and disease prevention.

### Keeping People Well Vs. Caring for the Sick

Health promotion comprises interventions intended to keep people well, as opposed to interventions designed to improve health once people are sick—essentially curative care. We recognize some overlap between prevention and curative care, but that has little or no effect on our assessment.

The key distinction between “sick care” and promotion of good health has long been recognized.^3^^–^^5^ Harvey V. Fineberg, President of the Institute of Medicine and former Dean of the Harvard School of Public Health for 13 years, contrasted traditional curative “sick care” and preventive approaches and gave reasons why prevention gets little attention (Box 1).

*In curative care, the principal professional responsibility is to the individual patient, whereas in preventive care, focus is often at the population level and entails a responsibility to the entire community. In curative care, solutions involve prescribing medication, performing operations, or delivering other clinical therapies; in prevention, there is a much wider array of possibilities, from changing behavior choices to altering social conditions, in addition to clinical interventions such as immunizations. Ensuring the health of a population is more difficult than delivering health care to an individual.*^6^

**BOX 1. Why Prevention Gets Little Attention**

Prevention all too often gets little attention, despite being culturally ingrained, as typified by Benjamin Franklin's proverb “an ounce of prevention is worth a pound of cure,” and although it is often highly effective and cost-effective. Harvey Fineberg, President of the Institute of Medicine, suggests that among these reasons are^6^:

- The success of prevention is invisible, lacks drama, often requires persistent behavior change, and may be long delayed.
- Statistical lives have little emotional effect, and benefits often do not accrue to the payer.
- Avoidable harm is accepted as normal.
- Preventive advice may be inconsistent, and bias against errors of commission may deter action.
- Prevention is expected to produce a net financial return, whereas treatment is expected only to be worth its cost.
- Commercial interests as well as personal, religious, or cultural beliefs may conflict with disease prevention.

### Health Promotion and Health Care Form Two Arms of the Health System

The 2 major “arms” of the health system over the life of an individual are:

1. Health promotion and disease prevention
2. Personal health care services (mostly curative)

The y-axis in the Figure represents health and illness; the x-axis is our full expected life span. Health care services are in the lower half of the figure, where individual clients who are ill receive personal health care, aiming to recover, as much as possible, their optimal health. Health promotion and disease prevention are in the upper half—generally population-based interventions provided to large numbers of people who are healthy.

**FIGURE:**
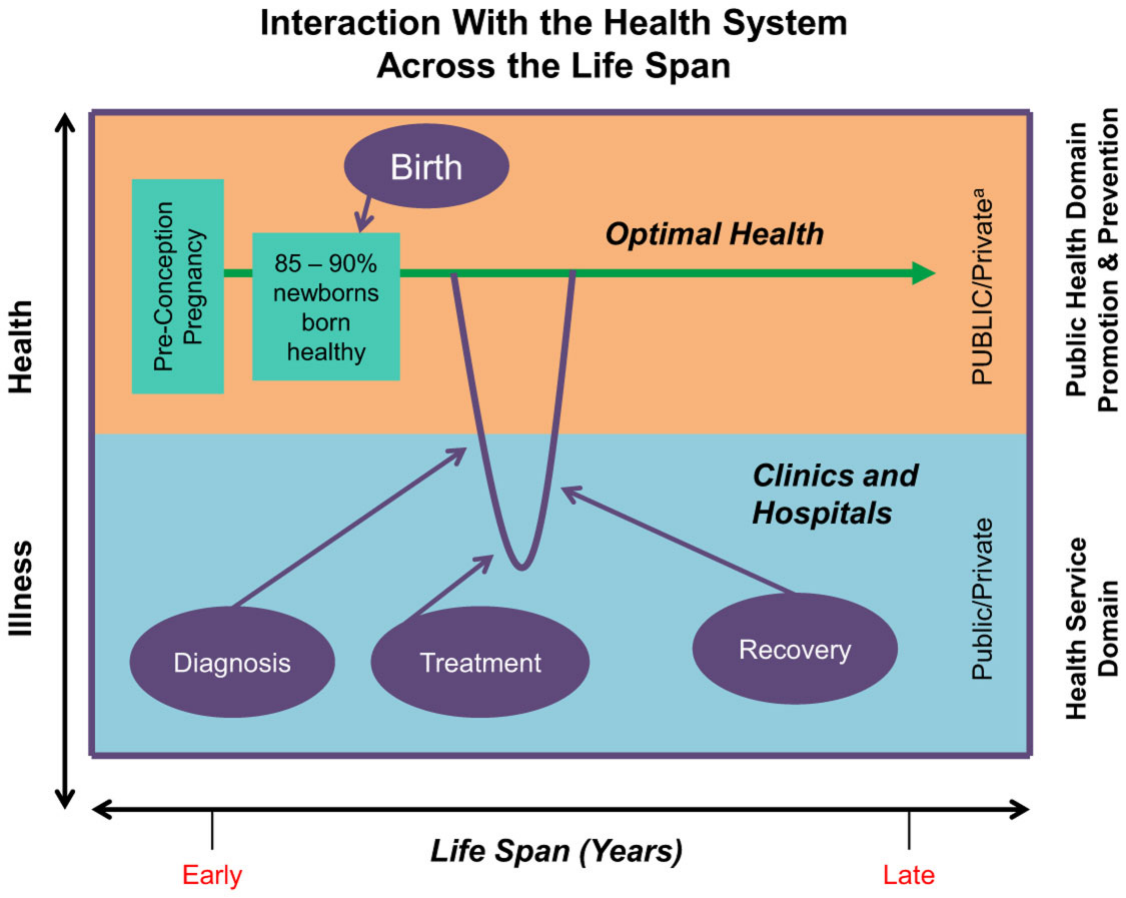
Two Major Arms of the Health System ^a^ Health promotion activities are considered public goods: goods that are available for the benefit of all, and where the use by one individual does not reduce availability to others. In contrast, with clinical care, the supply is limited by available resources, and provision to one person reduces the quantity available to others. Since public goods are available to all and it is difficult to charge or exclude users, they are undersupplied by private markets, making it essential for governments, or the public sector, to promote health and prevent illness. World Bank. Annual review of development effectiveness 2008: shared global challenges. Washington (DC): World Bank; 2008.

About 85% to 90% of newborns are born healthy (lower in developing countries), and, clearly, one role of the health system is to help keep infants, children, adolescents, and adults in optimal health across their life span. Should an individual become ill, s/he may need medical care including diagnosis and treatment that will lead to her/his recovery and hopefully to optimal health. The more effective and efficient the intervention to maintain health and prevent illness, the less the demand for (often costly) health care. Health promotion has a huge agenda, covering both infectious diseases and the emerging priorities related to NCDIs (Table).

**TABLE.** Major Health Promotion Categories for the Developing World

**Traditional Agenda**	**Emerging Agenda**
• Immunization • Family Planning • Breastfeeding • Undernutrition • Water and Sanitation • Safe Sexual Behavior • Bed Nets • Gender-Based Violence	• Tobacco • Alcohol • Overnutrition • Physical Activity • Salt Consumption • Drug Use • Injury

### Health Promotion Interventions Are Diverse

The following is a simplified list of health promotion interventions:

- “Medical” such as immunization, well-baby clinics, or the nicotine patch
- Persuasion or direct behavior change, such as mass media to promote breastfeeding
- Policy/regulatory, such as a tobacco tax or speed limits
- Environments and physical structures conducive to health, such as latrines, speed bumps, and standards for food service

The “medical” field of health promotion and disease prevention is being quickly and greatly transformed by the opportunities brought by the dramatic progress in genomic, proteomics, and pharmacogenomics. The potential to anticipate risks and individualize disease prevention and health promotion interventions is undeniable, although many technological and financial barriers still exist, particularly for low- and middle-income countries (LMICs). However, with the rapid progress in laboratory technology, the increased use of these laboratory resources, and the eventual reach of enough computational power, the accuracy, cost, and availability of these tests will no longer be a limitation. While many believe the widespread use of these methods will happen within the next 5 years,^7^ the other areas of health promotion in our simplified list will remain important.

Notice that many of these interventions lie outside the domain of clinical health services. Thus, they often call for diverse interventions, different expertise, and a population-level mindset. Behavior change and policy and legislative expertise are especially crucial.

## IMPACT OF PREVENTION ON CORONARY HEART DISEASE IN THE WEST

In the 1960s, the Framingham Heart Study provided information on the important role of exercise, diet, tobacco, and obesity on coronary heart disease (CHD).^8^ To reduce the impact of CHD, health promotion and disease prevention initiatives implement policies and programs promoting healthy behaviors and creating environments conducive to health. Among the more important programs reviewed in this article are those implemented in the Province of North Karelia, Finland (Box 2), in New York City (Box 3), and in the workplace by the private sector (Box 4). Such employer-based wellness programs provide a high return on investment.^21^^–^^23^ Employers invest generally around US$100 to $150 per participating employee each year; the health benefits are generally realized 3 years into the program. A 2008 literature review identified return on investment (ROI)* of worksite health promotion programs in the range of US$3–$10 for every $1 invested^24^; the European Agency for Safety and Health at Work estimates ROI 2.5–4.8 Euros per Euro invested.^25^

Employer-based wellness programs provide a high return on investment.

**BOX 2. Health Promotion and Disease Prevention in North Karelia, Finland**

**Remarkable impact achieved:** The North Karelia Project started in 1972. By 2006, the mortality rate from CHD in men 35–64 years old in North Karelia had declined 85% and the cancer mortality rate by 67%, particularly for cancers related to tobacco use. The project increased life expectancy by 8–11 years over a 35-year period (1971–2006)—from 66.4/74.6 years in men/women to 75.8/82.8 years nationally, and from 64/72 years to 75/81 years in North Karelia—and became a blueprint for Finnish public health interventions.^9^^,^^10^

**What North Karelia did:** The project promoted healthy lifestyles and changes in risk factors associated with NCDs using a 2-pronged approach of community organization strategies and health promotion programs.^9^ To achieve optimal health, the focus was on specific behaviors to reduce fat and salt consumption, increase fruits and vegetables eaten, reduce smoking and alcohol use, and increase physical activity.^9^^,^^11^

Community organization strategies include:

- Plan, implement, and evaluate programming with the community
- Increase physical activity, harvest wild berries, lower cholesterol through competitions between villages, education of community opinion leaders
- Healthy meals at schools and workplaces
- Increase access to low-fat, nonfat, and low-salt products, and to fresh fruits and vegetables during the winter, in supermarkets

Health promotion programs include:

- Health journalism in the broadcast media, including televised smoking cessation programs
- Basic health services and health promotion for large stratum of population
- Development and marketing of healthy foods by the Finnish food industry
- Shift in agricultural subsidies from high-saturated fat dairy products to low-fat and vegetable-fat options
- Prohibition of tobacco advertising and enforcement of smoke-free areas
- Worksite health promotion, diet, and exercise

Most importantly, the project was supported by the Governor of North Karelia as well as provincial and national political, civic, and health leaders and the WHO.

**BOX 3. Health Promotion and Disease Prevention in New York City**

**Remarkable impact achieved:** In December 2011, New York City (NYC) Mayor Michael Bloomberg issued an invitation: “If you want to live longer and healthier than the average American, come to New York City.”^12^ Research on life expectancy in 3,147 independent cities and counties in the United States estimated that Manhattan's life expectancy rose 10 years between 1987 and 2009, the largest increase of any county. In addition, the other 4 counties that make up New York City were all in the top percentile. In contrast, national life expectancy increased by only 3.06 years over the same period.^12^

**What New York City did:** In the early 2000s, the NYC health department began strong innovative programming through policy change and improved health care.^13^ Key actions were to:

- Discourage smoking by increasing the tax on cigarettes from US$0.08 to $1.50/pack, prohibiting smoking in indoor workplaces, offering smoking cessation clinics, and distributing nicotine gum and patches
- Improve nutrition and physical activity by requiring restaurants to phase out trans fats, establishing standards for food service, and developing safe places for physical activity, such as cycle lanes^14^
- Improve child health through home visits by nurses every 1–2 weeks to high-risk first-time mothers during pregnancy and for 2 years after birth
- Control communicable diseases through widespread condom distribution, and set up a drug overdose prevention program^15^
- Promote healthy behaviors through communication campaigns and public policies,^16^ and increase health care insurance coverage

Most importantly, Mayor Bloomberg consistently provided the enlightened robust political leadership needed for city-wide progress in public health.

**Future plans:** The NYC health department created a Division of Health Promotion and Disease Prevention with increased focus on information, communication, data to track impact, and sound policy with the vision of keeping an increasingly larger proportion of the population in optimal health.^13^

**BOX 4. Health Promotion and Disease Prevention by the Private Sector**

For more than 30 years, progressive employers have invested in health promotion wellness programs in the workplace.^17^

**Remarkable benefits achieved for employees**^**18**^^,^^**19**^**:**

- Increased well-being, self-image
- Improved health status and reduced health risk factors
- Reduced out-of-pocket expenses for health care and medication
- Enhanced job security and satisfaction
- Avoided disability

**Benefits for employers**^**18**^^,^^**20**^**:**

- Lowered absenteeism costs to employers by about US$2.73 for every dollar spent on wellness programs
- Enhanced employee productivity
- Decreased losses from illness and injuries
- Enhanced corporate image
- Improved employee recruitment and retention
- Lowered health care costs by about US$3.27 for every dollar spent on wellness programs

Given that per capita incomes and health care costs are much lower in developing countries than in the United States, it would require a much smaller investment by developing-country employers to implement a similar program, suggesting a higher return. On the other hand, some of the benefits in developing countries might be lower, reflecting the lower productivity compared with U.S. employees, although other benefits might be greater. On balance, it is probably reasonable to assume that the return on investment in developing countries would be at least as great as in the United States and could possibly be greater.

### Mathematical Modeling of Results of Primary Prevention Efforts

Results of mathematical modeling^26^ of risk factor reduction in apparently healthy people (primary prevention) and risk factor reduction in patients with CHD (secondary prevention) undertaken in England and Wales,^27^ Europe,^28^ New Zealand,^29^ and the United States^30^ show very powerful effects of primary prevention, equal to or greater than the reduction in deaths achieved by secondary prevention.

Mathematical modeling shows very powerful effects of primary prevention.

**England and Wales**^**27**^**:**

- CHD mortality has halved since 1981 in the United Kingdom, resulting in 68,230 fewer deaths in 2000.
- Current government initiatives favor risk factor reduction in CHD patients (secondary prevention), but population-based primary prevention might be more powerful.
- Approximately 45,370 fewer CHD deaths were attributable to reductions in smoking, cholesterol, and blood pressure in the whole population.
- Some 36,625 (81%) of these fewer deaths occurred in people without recognized CHD and 8,745 (19%) in CHD patients.
- Compared with secondary prevention, primary prevention achieved a 4-fold larger reduction in deaths.

**Europe**^**28**^**:** The risk of developing a chronic disease decreased progressively as the number of healthy factors increased, after adjusting for age, sex, educational status, and occupational status.

**New Zealand**^**29**^**:** In Auckland, approximately half the reduction in CHD mortality rate was attributed to medical therapies and half to reductions in major risk factors.

**United States**^**30**^**:** There were 745 deaths among the 8,375 people aged 20 years and older who participated in the 1999–2002 U.S. National Health and Nutrition Examination Survey, who were followed through 2006. Deaths were most likely to have occurred among study participants with no healthy behaviors; the more healthy behaviors people had, the lower their risk of being among those who died.

**All-Cause Mortality**^**31**^**:** A 2012 meta-analysis of 15 prospective studies on the combined effects of healthy lifestyle behaviors on all-cause mortality comprised 531,804 people with a mean follow-up of 13.24 years. The meta-analysis found that the more healthy lifestyle behaviors people had, the lower the risk of all-cause mortality. None of the studies included in the meta-analysis were conducted in LMICs; 7 were conducted in Europe, 5 in the United States, 2 in Japan, and 1 in China. Healthy lifestyle was defined as not or never smoking, optimal weight, physically active, a healthy diet, and moderate consumption of alcohol—behaviors relevant to both CHD and NCDs.

The mathematical modeling and meta-analysis confirm the importance, effectiveness, and cost-savings of population-based health promotion and disease prevention programs to facilitate optimal health of large numbers of people across their life span.

## SUCCESSFUL HEALTH PROMOTION IN THE DEVELOPING WORLD

Health promotion and disease prevention programs and interventions to increase the health and well-being of their populations are growing in the developing world. We highlight programs in Malaysia and Thailand as well as 4 global efforts: against tobacco, to promote physical activity, against obesity, and to promote healthy settings.

Health promotion programs are growing in the developing world.

### Malaysian Health Promotion Board

The Malaysian Health Promotion Board^32^ is a statutory body in the Ministry of Health established by an Act of Parliament in June 2006. It is governed by an independent body with members from relevant ministries, nongovernmental organizations (NGOs), and health promotion professionals. Its primary role is to define “the health promotion agenda across different sectors and settings,” primarily through building capacity and funding grants.

The priority areas for health promotion are:

- Prevention and control of tobacco and alcohol
- Promotion of healthy lifestyles, including exercise and healthy eating
- Environmental health
- Mental health
- Prevention of cancer, diabetes, cardiovascular disease, and obesity
- Sexual health, including HIV/AIDS
- Research
- Promoting health through sport, cultural, and arts activities

#### Malaysia's Remarkable Achievements

Among the results of the Smoke-Free Initiative, a survey of youth (13–17 years) and adult smokers and nonsmokers, found^33^:

- Since the 2004 smoke-free regulations came into force, there has been a doubling of bans against smoking in the workplace between 2005 and 2009, as reported by smokers who work indoors.
- The percentage of smoke-free homes increased dramatically from 7% in 2005 to 40% in 2009, as reported by adult male smokers.
- Three of four smokers support stronger government control of tobacco, even if it means paying more for cigarettes.
- Forty-three percent of smokers and quitters said that antismoking campaigns made them more likely to quit smoking or to stay tobacco free.

### Thai Health Promotion Foundation

ThaiHealth,^34^ an independent state agency established in 2001, is funded by a 2% tax on tobacco and alcohol, yielding an annual budget of about US$100 million; its Governing Board is chaired by the Prime Minister. ThaiHealth works with multiple partners and funds more than 1,000 projects each year. It is a member and Chair (2010–2012) of the International Network of Health Promotion Foundations that works to enhance the performance, and support the establishment of health promotion foundations based on innovative financing.

A 10-year review of ThaiHealth^35^ (2001–2011) identified the following achievements and challenges resulting from policy initiatives such as tobacco- and alcohol-free zones; behavior change communication campaigns coordinated with religious, community, and civil society leaders; and creating environments conducive to health.

#### Thailand's Remarkable Achievements

- **Tobacco:** Between 1991 and 2009, the number of smokers was reduced by 12.26 million.
- **Alcohol:** Between 2008 and 2009, sales of beer and whisky dropped by 178 million liters, reducing domestic expenditures by almost 8%.
- **Road safety and accident prevention:** Fatal accidents declined from 22.9/100,000 in 2003 to 16.82/100,000 in 2010.
- **Sports and physical activity:** Weekly physical education in schools increased from 1 to 2 hours, and 15% of the budget for this effort is for disabled persons and disadvantaged groups.
- **Child health:** In 2008, schools became carbonated-beverage free, and a ban was instituted on adding sugar to formula and supplemental foods for infants and young children.

#### Challenges

- Maintain and extend relevance of strategic health promotion interventions
- Develop rigorous approach to evaluation, and connect learning, knowledge, and capacity building
- Ensure process and procedure for granting awards are scrupulously fair, clear, and transparent
- Focus more on disadvantaged population groups

### Global Efforts Against Tobacco

The Framework Convention on Tobacco Control entered into force in 2005, reflecting broad consensus and strong evidence on effective, cost-effective interventions to reduce the massive and growing burden of disease caused by tobacco products.^36^ Powerful, practical summaries of the evidence and guidance provided by the World Bank,^36^^,^^37^ WHO,^38^ and other groups, and committed effective action by Australia, Brazil, Canada, South Africa, Thailand, and other countries, have enabled some countries to:

- Raise taxes to make tobacco products progressively less affordable
- Reduce tobacco product advertising
- Educate people—especially smokers through graphic labeling on cigarette packs—on the risks of use and benefits of quitting
- Reduce exposure to cigarette smoke by ensuring smoke-free public places
- Offer effective support to people who want to quit

The impact of determined, evidence-based antismoking measures is clear. A recent estimate finds that 41 countries that implemented, between 2007 and 2010, at least 1 of 5 key interventions to a level that met WHO criteria, will have reduced the number of smokers by 14.84 million and averted an estimated 7.42 million smoking-attributable deaths (range: 4.6–10.4 million) among smokers alive in 2007.^39^ There are other concomitant benefits, too—for example, Turkey cut smoking-related hospital admissions by 20% over 8 years.^40^

### Global Efforts Promoting Physical Activities

Regular physical activity is important for health. The benefits include weight control; stronger bones and muscles; longer life expectancy; and lower risk of cardiovascular disease, type 2 diabetes, and some cancers.^41^

Governments in Brazil, Canada, Chile, Colombia, France, Mexico, the United States, among many others, actively promote physical exercise through “Ciclovia” bicycle programs that are funded primarily by public funds.^42^ In general, Ciclovias use existing streets that are temporarily closed to motorized vehicles during weekends and holidays, although some cities develop special bicycle lanes used throughout the week.^43^ Cost-benefit ratios of 4 Ciclovia programs were 3.23–4.26 in Bogota, 1.83 in Medellin (both in Colombia); 1.02–1.23 in Guadalajara, Mexico; and 2.32 in San Francisco.^44^ The programs have strong popular support.

### Global Efforts Against Obesity

Brazil, Chile, Colombia, Costa Rica, and Peru^45^ have passed laws that focus on improving healthy food choices offered to children, generally in schools. Some countries control the advertising of food in schools, regulate food labeling, and limit advertising especially on television.

Brazil's approach to promoting healthy food in schools includes setting nutrition standards and requiring that 30% of food in school meal programs be locally grown or manufactured and 70% be unprocessed.^45^

Peru has implemented nutritional education in schools, information campaigns by the education and health ministries, monitoring nutrition, overweight, and obesity among children and adolescents, healthy food in school kiosks or cafeterias, more physical activity, and controls on advertising aimed at children and adolescents younger than 16 years.

In Mexico, one of the most obese nations according to a United Nations report,^46^ legislators approved an 8% tax on soft drinks and high-calorie foods such as fried foods and sweets.^47^ Mexican lawmakers are also requiring 30 minutes of daily physical education classes in schools.^48^

### Global Efforts Promoting Healthy Settings: Cities, Municipalities, Communities

“Healthy Settings” is a strategy supported by WHO to implement intersectoral health promotion partnerships including local governments to create environments conducive to health.^49^ “Healthy Cities,” among the best-known and largest of the settings approaches launched in the 1980s, is defined as a city that is:

*continually creating and improving those physical and social environments and expanding those community resources which enable people to mutually support each other in performing all the functions of life and developing to their maximum potential.*^51^

The movement is considered a success. Thousands of cities around the world are implementing Healthy Cities partnerships, generally involving distal interventions, such as community organization and social development, organizational and infrastructure development, and policy development. The focus of this collaborative action often includes bike lanes and safe public space for walking and other physical exercise, better public transport, smoke-free public spaces and other pollution reduction, and healthy and affordable housing. Evaluations of the Healthy Cities project in Europe found that an appropriate mix of distal interventions for health provides a broad and sustainable effect on population health, whereas proximal interventions (for example, health and patient education, health care) yield focused health gains (often disease, gender, and age-group specific) at relatively high cost.^51^^,^^52^

## STATUS OF HEALTH PROMOTION AND DISEASE PREVENTION IN AFRICA

In 2001, the Member States of the World Health Organization Regional Committee for Africa (WHO AFRO) approved a health promotion strategy “to foster actions that enhance physical, social and emotional well-being.”^53^ Among the activities suggested were to provide technical support and capacity-building workshops and to form partnerships and alliances. In its review of the 10-year progress report,^54^ WHO AFRO recognized that implementation of the regional health promotion strategy revealed several gaps and challenges:

- Weak leadership and stewardship among ministries of health to coordinate health promotion activities across sectors
- Low level of involvement of various players, including civil society and communities, in advocacy actions to regulate and legislate for good health governance
- Inadequate evidence on the effectiveness of health promotion
- Lack of a sustainable financing mechanism
- The need to build a critical mass of health promotion practitioners, including at the community level

In view of these challenges, WHO AFRO passed the Health Promotion: Strategy for the African Region Resolution in November 2012.^55^ Among the objectives of the strategy is:

… to strengthen the capacity of Member States to develop, implement, monitor and evaluate health promotion strategies, policies, and regulatory and legislative frameworks that address the risk factors and the determinants associated with communicable diseases and non-communicable diseases, violence and injuries, maternal and child health conditions, and new and re-emerging threats to health.

The Consortium for Non-Communicable Diseases Prevention and Control in sub-Saharan Africa (CNCD-Africa),^56^ established in July 2009, aims to underscore the role of health promotion in addressing NCDs in Africa and to develop advocacy strategies for addressing NCDs using a health promotion approach. When asked about the state of health promotion in Africa, Jared Odhiambo Owuor, Executive Secretary of CNCD-Africa, replied:

*Most health systems in Africa operate from a reactive standpoint when responding to public health needs. This approach pushes health promotion to the fringes. There is currently a need for the reorientation of healthcare staff and the sensitization of the public on the benefits of health promotion, as well as the need to proactively work towards healthy outcomes.*^57^

In 2010, the African Centre for Global Health and Social Transformation and The New York Academy of Medicine with the support of The Rockefeller Foundation noted the relatively low investment in public health:

*In LICs [low-income countries], 70 to 80 % of the disease burden is attributable to preventable infectious diseases for which the most effective intervention is public health action. Despite this, less than 10% of national health expenditures … are invested in public population health services (according to national health accounts data)*.^58^

Among the challenges mentioned by Ministers of Health were:

We have lots of human resources, but they're poorly trained.

Health promotion and disease prevention present particular challenges, due to the traditional emphasis on direct medical services and development and maintenance of medical facilities.

[Ministries] struggled to effectively disseminate health messages to positively influence the public's value on health.

### Low Spending on Prevention and Public Health

To a degree, the shortfalls mentioned above reflect low spending for health promotion. A recent report^59^ analyzes trends in prevention and public health (PPH) expenditures from 2005–2010 in 16 African countries with National Health Accounts (NHAs).† (The 16 countries are: Benin, Burkina Faso, Botswana, Côte d'Ivoire, Democratic Republic of the Congo, Ethiopia, Kenya, Liberia, Mozambique, Malawi, Namibia, Nigeria, Rwanda, Senegal, Tanzania, and Zambia.)

Many African countries spend little on health promotion.

Across the 16 countries, PPH expenditures per capita vary from US$4 in Burkina Faso to US$64 in Botswana. In 13 of the 16 countries, spending for curative care accounts for the largest percentage of total health expenditures (THE); in 9 countries, PPH services are the second largest expenditure. Expenditures for prevention of NCDs account for less than 3% of THE in every country. Only 9 countries provide data on how PPH expenditures are spent: most goes for prevention of communicable diseases, and about 10% is classified as “other.”

Data on the financing agents of PPH, defined as institutions or entities that channel funds provided by financing sources to pay for, or purchase, health services,^60^ are available in 13 countries. In 4 countries, the primary financing agent managing PPH expenditures is the government, in another 8 it is NGOs (both local and international) and donors.

Data on providers of PPH services are available in 8 of the 16 countries. In 6 countries, providers of PPH programs received a majority (45% to 96%) of PPH expenditures, while in 2 countries the majority of PPH expenditures are used by ambulatory care providers.

Ministry of Health policy and decision-makers in the African Region recognize the importance of implementing health promotion programs to “foster actions that enhance physical, social and emotional well-being.”^53^ To achieve this important goal, the countries also recognize they need undergraduate and graduate educational programs and highly specialized personnel. Yet the National Health Accounts indicate low per capita spending for PPH for NCDs by LMICs, compounding future challenges for health systems to maintain their populations in optimal health.

## HEALTH PROMOTION: A CALL FOR SPECIALIZED SKILLS FOR HUMAN RESOURCES

As demonstrated by the case studies reviewed in this article, ensuring healthy populations across their life span requires government to implement integrated health promotion and disease prevention interventions in the 5 action areas suggested in the Ottawa Charter^4^:

1. Build healthy public policy
2. Create supportive environments
3. Strengthen community action
4. Develop personal skills
5. Reorient health services

Health promotion and disease prevention is achieved by persuading, enticing, and requiring people to adopt healthy behaviors, and by changing environments to enable and be conducive to healthy individual and collective behaviors. Among the goals of health promotion is to ensure a health-literate society, with the “ability to obtain, process and understand basic health information and services needed to make appropriate health decisions and follow instructions for treatment.”^61^

Three groups have important roles in health promotion and disease prevention:

1. Policy makers and practitioners in health and other sectors (especially finance, infrastructure, and education) that have opportunities to adopt and implement health-promoting policies and actions
2. Specialists in health promotion and disease prevention
3. Health care providers who need to mainstream health promotion and disease prevention in their health care
  Policy makers, health promotion and disease prevention specialists, and health care providers have important roles in health promotion programs.

A stronger cadre of health promotion personnel could support and guide their colleagues working in nutrition, maternal and child health, family planning, mental health, tuberculosis, malaria, dental health, emergency care, and others areas.

Physicians, nurses, and others health sector workers need far more understanding of how they can promote health in the course of all their patient contacts. Typically, medical training gives this scant attention, despite the evidence that, for example, even brief interventions by physicians can have a significant impact on smoking behavior.

The specialized skills needed in health promotion and disease prevention programs include 5 areas:

- **Legislation and health policy:** the ability to analyze health promotion, disease prevention, and health care from the health policy perspective and to identify the policies and interventions most likely to achieve a given set of goals
- **Social and behavior change communication:** the ability to plan, implement, and evaluate persuasive communication programs to change individual and collective behaviors; increase awareness, knowledge, perception of risk, confidence to take action, and intention to act; create more favorable attitudes; influence social norms and policies; and improve interpersonal communication skills of health professionals
- **Prevention and community health:** the ability to empower community leaders, create awareness, and stimulate community commitment to health and action, to ensure community health services and programs meet community needs
- **Health journalism:** the ability to regularly place information on health at no cost in broadcast, print, and online media, and training for health care personnel to become media spokespersons
- **Multisectoral health promotion:** the ability to work at high levels of government and with NGOs and the private sector to address important risk factors influencing national and sub-national epidemiological profiles
  Specialized skills needed in health promotion programs include: legislation and health policy, social and behavior change communication, community health, health journalism, and multisectoral health promotion.

Important areas of interministerial action include:

- Ministry of Finance to set taxes to make tobacco and other harmful products progressively less affordable
- Ministry of Education to develop age-appropriate health content for primary and secondary schools, ensure healthy schools, healthy meals, and physical education programs; include health promotion and disease prevention theory, methods, and practices in the curricula of schools of medicine, nursing, dentistry, and allied health professionals and paraprofessionals
- Ministry of Labor and Commerce to promote healthy smoke-free workplaces, promote healthy options at workplace cafeterias, and push the food industry to provide healthy products
- Ministry of Transportation to ensure safe road construction, especially for pedestrians and cyclists, car safety, and safe driving
- Ministry of Communication to prohibit tobacco advertising and promotion, and sexual and violent content in programming for children
- Private sector, such as restaurants, to include healthy menu options, limit excess salt and fat, and provide smoke-free environments

## CONCLUSION

UHC will be financially feasible only when governments implement robust evidence-based health promotion programs, staffed by a cadre of professionals with specialized skill sets and appropriate levels of financial and political support to implement broad, effective programs. Effective health promotion and disease prevention makes economic sense, relieves pressure on the clinical health system, and directly improves people's well-being and optimal health across the life span. We urge developing:

1. Curriculum in health promotion and disease prevention in schools of public health that encompass broad specialized skills
2. More promotion and prevention in curricula of medical, nursing, and allied health professional schools
3. Clear job profiles and attractive salary levels for health promotion and disease prevention professionals
4. Legal, operational, and management frameworks for health ministry health promotion units (national and sub-national), including clear roles and responsibilities and adequate budgetary allocations
  To be financially feasible, universal health coverage requires robust evidence-based health promotion programs.

## Footnotes

* To estimate the ROI, baseline information on health costs are taken before the wellness program begins and again 3 years later, and a comparison is made between health costs of participating employees and of those that are not participating; the difference provides an estimate of the return. The experience of U.S. employers is that about half of employees participate in company wellness programs.

† National Health Accounts is a standardized framework for tracking health sector financial resource flows from sources, to agents that manage funds, to providers and consumers of care. Health promotion and disease prevention expenditures are classified as prevention and public health in this framework.
